# Evaluating the performance of infectious disease forecasts: A comparison of climate-driven and seasonal dengue forecasts for Mexico

**DOI:** 10.1038/srep33707

**Published:** 2016-09-26

**Authors:** Michael A. Johansson, Nicholas G. Reich, Aditi Hota, John S. Brownstein, Mauricio Santillana

**Affiliations:** 1 Dengue Branch, Division of Vector-Borne Diseases, Centers for Disease Control and Prevention, San Juan, Puerto Rico; 2 Center for Communicable Disease Dynamics, Harvard T. H. Chan School of Public Health, Boston, Massachusetts, USA; 3 Department of Biostatistics and Epidemiology, University of Massachusetts, Amherst, Massachusetts, USA; 4 Computational Health Informatics Program, Boston Children’s Hospital, Boston, Massachusetts, USA; 5 Department of Pediatrics, Harvard Medical School, Boston, Massachusetts, USA; 6 J.A. Paulson School of Engineering and Applied Sciences, Harvard University, Cambridge, MA, USA

## Abstract

Dengue viruses, which infect millions of people per year worldwide, cause large epidemics that strain healthcare systems. Despite diverse efforts to develop forecasting tools including autoregressive time series, climate-driven statistical, and mechanistic biological models, little work has been done to understand the contribution of different components to improved prediction. We developed a framework to assess and compare dengue forecasts produced from different types of models and evaluated the performance of seasonal autoregressive models with and without climate variables for forecasting dengue incidence in Mexico. Climate data did not significantly improve the predictive power of seasonal autoregressive models. Short-term and seasonal autocorrelation were key to improving short-term and long-term forecasts, respectively. Seasonal autoregressive models captured a substantial amount of dengue variability, but better models are needed to improve dengue forecasting. This framework contributes to the sparse literature of infectious disease prediction *model evaluation*, using state-of-the-art validation techniques such as out-of-sample testing and comparison to an appropriate reference model.

Dengue is a substantial public health problem in most of the tropical and subtropical regions of the world^1^. In most of these areas, dengue is endemic with cases occurring year-round, yet there is marked variation in incidence of dengue both within and between years. In Mexico, for example, yearly reported incidence over the last few decades has varied from several thousand cases to over 100,000 cases in 2009^2^. Even understanding the current burden of dengue can be challenging. There is often an extended delay between symptom onset and official reports reflecting care-seeking behavior or confirmed clinical reports of illness. In some settings, complex, multi-tiered reporting systems may contribute to delays. Because of the vastly greater burden of disease in larger epidemic years and difficulty in understanding current and future needs, much effort has been placed on developing early warning systems to predict or detect large epidemics as early as possible with the hope of being able to control epidemics in their early stages^3^,^4^.

Numerous mechanistic models have been developed to use detailed knowledge of dengue virus transmission biology to predict the evolution of dengue epidemics^5^,^6^. However difficulties emerge when attempting to use mechanistic models for forecasting, often the key mechanistic assumptions are unclear and the data needed to parameterize and feed the model are often difficult or impossible to obtain.

Models for large-scale dengue early warning systems have therefore mostly focused on two components, temporal autocorrelation and an association with weather or climate. Temporal autocorrelation results from the infectious nature of dengue viruses; cases are more likely in the near future when the current prevalence of infection is high. The influence of weather is due to the mosquito vectors of dengue viruses, *Aedes aegypti* and *Ae. albopictus*. Temperature, humidity, and precipitation are important determinants of mosquito reproduction and longevity^7^,^8^,^9^, and temperature has a strong influence on the ability of the mosquitoes to transmit dengue viruses^10^.

These two components, autocorrelation and weather or climate, form the basis for a wide variety of efforts to predict dengue incidence in countries such as Australia^11^, Bangladesh^12^, Barbados^13^, Brazil^14^,^15^,^16^,^17^, Colombia^18^,^19^,^20^, Costa Rica^21^, China^22^, Ecuador^23^, Guadeloupe^24^, India^25^, Indonesia^26^,^27^, Mexico^28^, New Caledonia^29^, Peru^30^, Philippines^31^,^32^, Puerto Rico^33^, Singapore^34^,^35^,^36^,^37^,^38^, Sri Lanka^39^, Taiwan^40^,^41^, Thailand^42^,^43^,^44^,^45^,^46^, and Vietnam^47^.

In this manuscript, we focus on developing prediction models for dengue incidence in Mexico based on observed dengue incidence and weather. We focus explicitly on building models that directly predict dengue incidence rather than those designed to classify future transmission, for example as low or high^29^,^30^,^34^,^42^. Although classification may be more useful for public health decision-making, the classification process is subjective^48^, making estimation of uncertainty a less straightforward process compared to direct prediction of incidence.

Autoregressive integrated moving average (ARIMA) models^49^ have been used extensively in dengue prediction efforts^14^,^18^,^19^,^35^,^37^,^40^,^45^, often incorporating a seasonal component (SARIMA)^11^,^14^,^15^,^24^,^25^,^27^,^43^,^46^,^47^. We used the SARIMA framework to define a dynamic suite of prediction models for dengue in Mexico that is at once flexible and wide-ranging, while remaining manageable in size for the purposes of careful evaluation and comparison. While the fundamental biology leading to autocorrelation and associations with weather and dengue incidence is clear, the specific contributions of these two components to dengue prediction remains less so. In other words, we wanted to answer the question, to what extent does incorporating climate data into models improve dengue forecasts at different spatial scales? Therefore, we assessed three specific features of these dengue prediction models: (i) the most important autoregressive and climatological components for predicting dengue incidence; (ii) the variability in importance of these components across different geographical areas; and (iii) the limits of prediction accuracy across models at different time horizons. Another important motivation of this study was to establish a forecast assessment framework that can serve as a reference for any infectious disease forecasting problem, including comparison to a non-naïve baseline model and validation on completely out-of-sample data.

## Materials and Methods

### Data

The number of dengue and dengue hemorrhagic fever cases reported for each month from January 1985 to December 2012 was obtained from the Mexican Health Secretariat^2^. To model these counts as a linear process, we log-transformed the highly skewed monthly dengue cases after adding one to the observed count. This eliminates computational problems associated with taking the log of zero, and can be thought of as accounting for the potential entrance of new cases^50^. Weather data from January 1985 to December 2012 were obtained from the National Oceanic and Atmospheric Administration North American Regional Reanalysis dataset (www.esrl.noaa.gov/psd/ accessed on May 1^st^, 2013). For each month and state, we extracted the average temperature (°C), daily precipitation (mm), and relative humidity (%).

### Models

We analyzed three different types of temporal models: linear models, autoregressive (AR) models, and seasonal autoregressive models (SAR). We label these models as (*p*,*d*,*q*)(*P*,*D*,*Q*)_*s*_ + *covar*_*L*_. For the ARIMA component, (*p*,*d*,*q*), *p* indicates the autoregressive order, *d* indicates differencing, and *q* is the order of the moving average^49^. For the seasonal component, (*P*,*D*,*Q*)_*s*_, *s* indicates the season length (*s* = 12 months for the monthly data presented here), *P* indicates the autoregressive order, *D* indicates differencing, and *Q* is the order of the moving average. The final component,  + *covar*_*L*_, indicates a particular covariate at a lag of *L* months included as a linear regression term.

We used a systematic procedure to select SARIMA models to fit to the data and evaluate for predictive performance. Each time series was assessed for stationarity using the Augmented Dickey-Fuller test^51^. We used domain-specific knowledge about infectious disease models to create a limited model space that we could explore fully and justify scientifically. Defining the model space in this way mitigated the risk of overfitting the model in the training period. Specifically, we chose to focus on models that (a) included lagged observations of up to three months (p = 1, 2, or 3) and three years (P = 0, 1, 2, or 3), (b) included differencing terms of up to order 1 (i.e. d or D = 0 or 1), and (c) excluded all consideration of moving averages (i.e. q and Q both fixed at 0).

Prediction models that include a differencing term (i.e. d = 1 or D = 1) have a term added to the model formula that captures the difference between the lag-1 and lag-2 case counts. These models therefore incorporate information about the most recently observed slope of dengue incidence: e.g. are reported cases increasing or decreasing? In some formulations, this can be considered a crude approximation to the reproductive rate of the disease, an important parameter for judging the trajectory of the outbreak^52^. As an example of a formula for a model including differencing, a (1,0,0)(2,1,0)_12_ multiplicative SARIMA model can be expressed as:


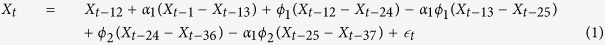


where 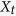 are the observed numbers of cases of dengue at time 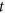; 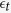 are the residual error terms, assumed to be normally distributed; and the coefficients 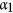, 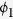, and 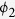 are determined to minimize the residual squared error.

To evaluate the added predictive value of weather variables, we examined the best SARIMA models from the model space defined above by adding individual weather variables as covariates, with lags of 1, 2, and 3 months and the month with the maximum Pearson correlation with dengue incidence (labeled with the sub-script *MX* in the figures), and assessing the change in predictive performance. Our aim was to single out the effect of each variable (precipitation, relative humidity, and temperature). Additional models including multiple weather variables at once were considered. Ultimately, the models presented represent a given SARIMA model plus the most highly correlated lagged weather covariate. All models were fitted in R^53^, using the arima function from the stats package.

### Model evaluation

The data was separated into three subsets. The first five years of data (1985-1989) was reserved for model training. Models trained on that data were used to dynamically predict dengue incidence over an 18-year model evaluation period (1990–2007). The final five-year dataset (2008–2012) was reserved for a complete out-of-sample validation after model selection.

At the end of the training period, predictions were made for the next 1–6 months (e.g., predictions are made for January–June based on data through December of the preceding year). Then the model was refitted with data from the next month (i.e., January) and predictions were made for the following 1–6 months (February–July). This process was repeated over all months of the evaluation period. Predictions were evaluated by comparing observed incidence with model-predicted incidence using two metrics: mean absolute error (MAE) and the coefficient of determination (R^2^). For comparisons of predictions across different locations and predictions horizons, we calculated the change in MAE relative to the best prediction among all models for a given location and horizon:


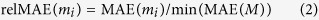


where *m*_*i*_ is a single model in the set of models *M* for a given time and location. Thus the model with the best point prediction has relMAE = 1 and predictions further from the observations will have increasing relMAE values.

To compare two specific models (*m*_1_ and *m*_2_), we calculated the relMAE:


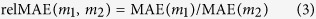


### State-level models

We systematically assessed the predictive performance of a wide range of models at different spatial scales, using data aggregated at the national level and at the level of states. The primary interest of this manuscript is to assess models for forecasting dengue incidence in endemic locations, so we restricted the analysis to the 17 states reporting cases in at least half of the months in the training and development time period (1985–2007). Some of the other states, such as Sonora, Coahuila, and San Luis Potosi, had high median annual incidence over this period due to sporadic outbreaks but reported cases in less than 50% of the months.

Using the selection algorithm described briefly above in the “Models” subsection and in more detail below in the “National dengue forecasts” subsection, we fitted and evaluated 39 models at each spatial scale. The same selection process was repeated for each location separately. We assessed the predictions of all models across each location using the average relative MAE and R^2^ for each prediction horizon. For each state we selected an optimal model for short-term forecasts by identifying the model with minimum average relative MAE across the 1–3 month prediction horizons, for each state we refer to this model as the “local” model. Assessing model performance across all spatial scales in the training period, we identified a single “common” model that performed well across all spatial scales. During the prospective out-of-sample forecast phase, after model selection, we compared the performance between the common and local models.

## Results

### National dengue forecasts

**Figure 1:**
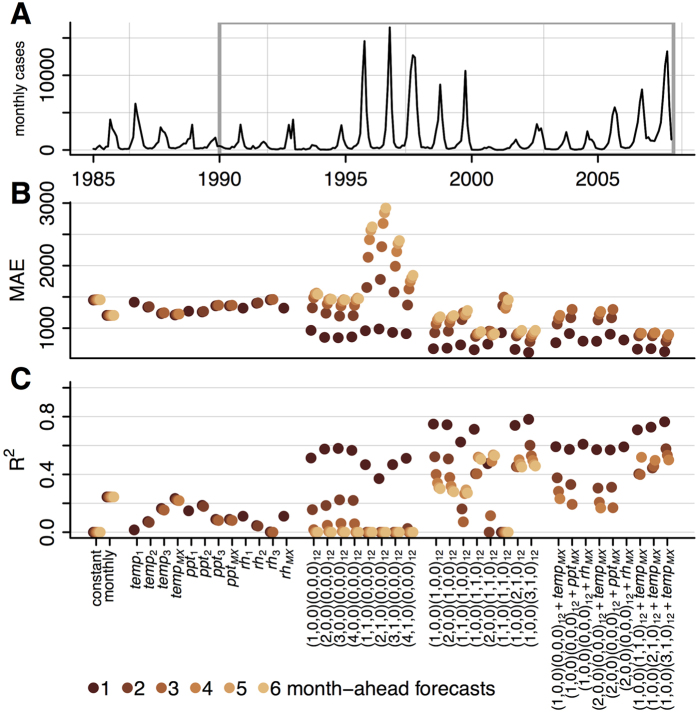
National-level forecast metrics. National-level incidence is shown during the training period (1985–1989) and evaluation period (1990–2007). (**A**) For each of 39 models considered, the MAE (**B**) and R^2^ (**C**) values for prospective forecasts over the entire evaluation period are shown for each prediction horizon (dark red to yellow, corresponds to 1–6 months). For models including lagged weather covariates, forecasts were not possible at prediction horizons beyond the lag and are not shown. An equivalent plot for each state is shown in Figure S1.

During the 5-year training period (1985–1989) and 18-year evaluation period (1990–2007) the monthly dengue incidence in Mexico showed strong seasonality (Fig. 1A). Incidence also varied substantially between years, with less than 2,000 cases reported in 2000 and more than 50,000 reported in 1997.

We first compared two naïve models for predicting national dengue incidence several months into the future, one estimating that future incidence follows the historical average incidence and another assuming that future incidence for a specific month will be the historical average for that particular month of the calendar year (Fig. 1B). These models were implemented at the beginning of the evaluation period, with predictions made for months 1 through 6 of that period. An additional month of data was then acquired (month 1), the model was refitted, and predictions for the next 6 months (i.e., months 2–7) were made, ensuring that all forecasts were made on out-of-sample data. As predictions were made at each month throughout the 18-year evaluation period, a total of 216 predictions were analyzed for each prediction horizon and each model. For all models, we used the same dynamically increasing training set strategy to calculate the respective out-of-sample predictions, strictly avoiding the use of *forward-looking* information. This allowed the use of predictive metrics to compare models directly.

Across all of these out-of-sample predictions, the model using the month of the year outperforms the long-term mean model by both MAE (lower error) and R^2^ (higher correlation). Despite being relatively naïve, predictions from the seasonal model have an R^2^ of approximately 0.24.

We then assessed twelve linear models including data on average temperature, daily precipitation, and relative humidity in previous months. The maximum cross-correlation between each of these weather variables and dengue incidence was 1 month for relative humidity, 3 months for precipitation, and 4 months for temperature. We assessed lags of 1–3 months for each variable and also a 4-month lag for temperature. The 4-month lag temperature model performed best, but did not outperform the simple monthly model by either metric.

Next we assessed sixteen models with only autocorrelation and differencing terms: eight models including only short-term autocorrelation and eight models including both short-term and seasonal autocorrelation. The first-order AR model (1,0,0)(0,0,0)_12_, substantially improved the 1-month predictions compared to all previous models. At 2-months, the predictions were less accurate than those from the monthly model and some of the covariate models. Increasing the order of the AR model slightly improved the predictions, while differencing decreased their accuracy. Adding a seasonal component markedly improved the predictions; the (1,0,0)(1,0,0)_12_ SAR model outperformed all of the previous models with lower MAE and higher R^2^ at all prediction times. Increasing the short-term AR component slightly decreased accuracy and 1-month differencing decreased accuracy substantially. Adding a seasonal differencing term however, improved accuracy by both metrics for longer prediction horizons (e.g. (1,0,0)(1,1,0)_12_). Increasing the order of the seasonal AR term to (1,0,0)(3,1,0)_12_ further improved predictions.

Finally, we assessed nine AR and SAR models with weather covariates. At all prediction horizons, models containing first order autoregressive terms and covariates consistently outperformed models containing only the covariate information (e.g. (1,0,0)(0,0,0)_12_+*temp*_*MX*_ versus *temp*_*MX*_) and simple AR models (e.g. (1,0,0)(0,0,0)_12_+*temp*_*MX*_ versus (1,0,0)(0,0,0)_12_). The SAR models with covariates have very similar accuracy to the SAR models without covariates at 1–4 month prediction horizons. For 5- and 6-month horizons, the covariate models could not make predictions as they extended beyond the 4-month lag for temperature. Additional analyses showed that once about 12 years of training data were used, the predictive performance of the models reached a stable average value (between 0.85 and 0.9 correlation), although year-specific values showed some variation around the average (data not shown). Finally, in a non-exhaustive search, we noticed that using all weather variables at once as input in a given model did not yield noticeable improvements.

Overall, the best performing models were (1,0,0)(3,1,0)_12_ for horizons of 1–2 months, (1,0,0)(3,1,0)_12_+*temp*_*MX*_ for the 3-month horizon, (1,0,0)(1,1,0)_12_ for the 4-month horizon, and (2,0,0)(1,1,0)_12_ for 5- and 6-month horizons (Fig. 1C). The best model across all horizons was (1,0,0)(3,1,0)_12_, followed by (1,0,0)(1,1,0)_12_ and (1,0,0)(2,1,0)_12_.

### State-level dengue forecasts

**Figure 2:**
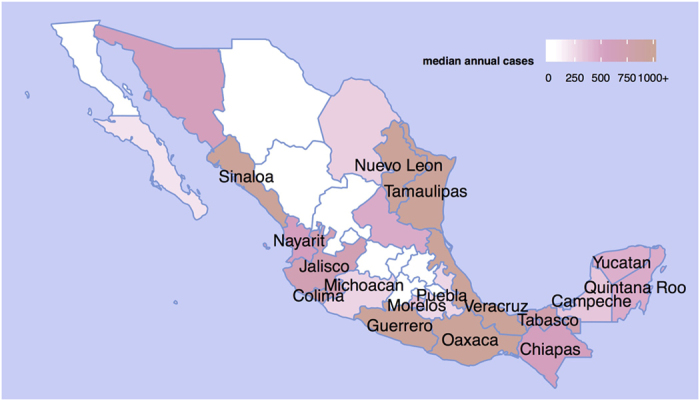
Map of included states. States with median monthly incidence greater than zero during the training and development periods (1985–2007) were selected for forecasting model development (labeled). This figure was produced using the statistical computing environment R, version 3.2.3 (http://www.R-project.org).

**Figure 3:**
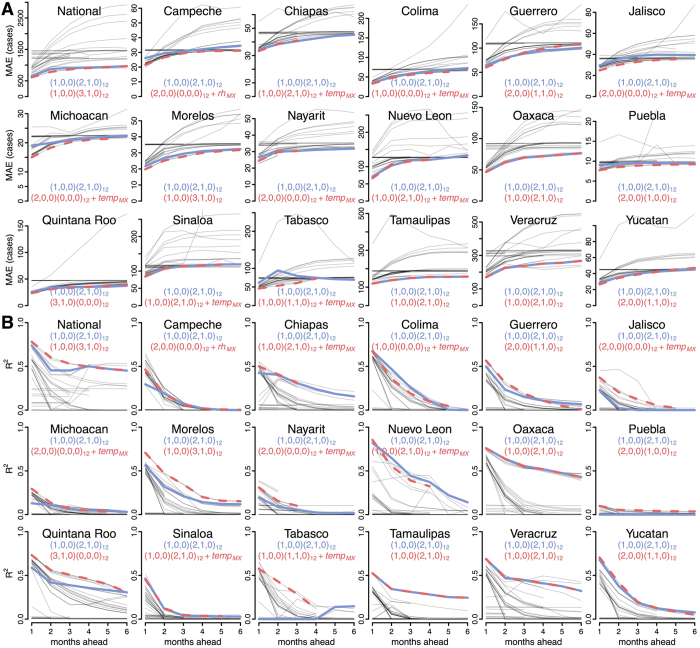
Forecasting metrics for all models in Mexico and 17 Mexican states. MAE (**A**) and R^2^ (**B**) values are shown for each of 39 models at every prediction horizon (grey lines). The optimum local (red, dashed) and common (blue, solid) models are superimposed. Full detail for all models is shown in Fig. 1 for all of Mexico and Figure S1 for each state.

Dengue incidence varies substantially between states within Mexico (Fig. 2). Similar to the national-level results, the first-order AR model (1,0,0)(0,0,0)_12_ outperformed the monthly model at shorter prediction horizons of 1–3 months (Fig. 3, Supplementary Figure 1). However across all horizons and states, the most accurate model was the (1,0,0)(2,1,0)_12_ model, which we now refer to as the “common” model. The coefficients for the (1,0,0)(2,1,0)_12_ model in each state varied, but the pattern was consistent across all states: the monthly autoregressive component was positive in each state and the seasonal autoregressive components were negative, with decreasing magnitude at longer lags (Supplementary Table). The next best model was (1,0,0)(3,1,0)_12_. Adding meteorological covariates to either of these generally resulted in increased error.

Nine of the seventeen local models, i.e. the models with minimum average relative MAE across the 1–3 month prediction horizons, included meteorological variables (Fig. 3). Post-evaluation period coefficients for the local and common models are shown in the Supplementary Table.

### Prospective forecasts

**Figure 4:**
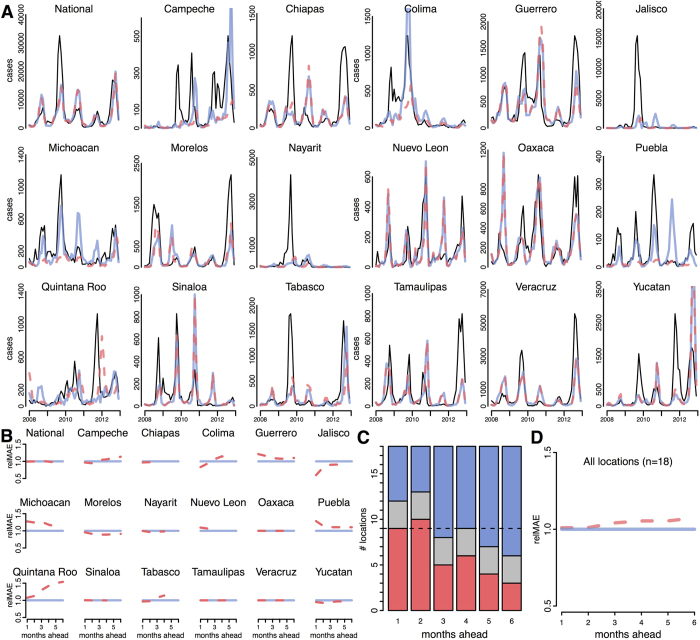
Prospective prediction evaluation over the 5-year validation period (2008–2012). The local (red) and common (blue) 3-month ahead forecasts are compared to reported dengue incidence (black, dashed lines) (**A**). The relative MAE compares the local model (red) to the common model (blue) over the entire 5-year validation period for individual locations (**B**). Values less than one indicate decreased error, i.e. improved performance, of the local model relative to the common model. Across all locations the number of locations in which the local model is more accurate than the common model declines at increasing prediction horizons (**C**) (red = local, blue = common, grey = the local and common models are the same). The relative MAE over all locations for the local model increases at longer prediction horizons.

At the national and state level we used the analysis described above to implement prospective forecasts for the validation period, 2008–2012, using the local and common models. Both models tended to capture seasonality and some of the inter-annual variability (Fig. 4A). However, they underestimated the magnitude on numerous occasions in different locations, particularly during the epidemic of 2010. This was most pronounced in Jalisco and Nayarit. Of the 18 time-series analyzed (17 state-level, 1 national-level), stationarity varied, with 7 potentially non-stationary states.

The relative performance of the two models varied by location and prediction horizon (Fig. 4B). In most locations, the local models were more accurate than the common model for predictions at 1–2 months (Fig. 4C). At 3–4 months, the accuracy was similar, and at 5–6 months the common model was more accurate. Generally, the difference between the two models was small. The local model was at least 10% more accurate than the common model at a total of 6 horizons in three states – Colima (1-month horizon), Jalisco (1–2 month horizons), and Morelos (3–5 month horizons). The common model was 10% better than the local model in seven states for a total of 17 prediction horizons. Taking the average of the differences across all locations (national and states), the common model was approximately 0.1% more accurate than the local model at 1- and 2-month horizons, with this difference increasing at longer horizons (Fig. 4D). Averaged across all locations, the common model was more than 4% more accurate at horizons of 3–6 months.

**Figure 5:**
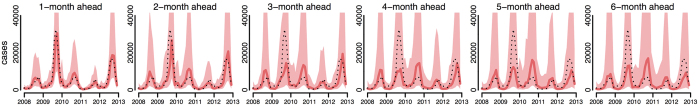
Prospective predictions with uncertainty. Forecasts from the national (1,0,0)(3,1,0)_12_ model with 95% Confidence Intervals are compared to reported dengue incidence (black, dashed lines). The (1,0,0)(2,1,0)_12_ model and models for each state are shown in Figure S2.

Finally, we assessed the uncertainties in predictions (Fig. 5 - national, Supplementary Figure 2 – national and states). Forecasts for 1-month horizons had some ability to confidently distinguish a low season from a high season, and to a lesser extent at a 2-month horizon. However at 3-months and beyond, the expected number of cases in the peak transmission season generally ranged from zero or a very low number of cases to more cases than have ever been reported for both the local and common models.

## Discussion

Increasing data availability and novel analytical tools have led to a growth in research on infectious disease forecasting. As these efforts move towards real-time implementation for use by public health decision makers, it is vital that we move towards a common understanding of how forecasts and the accompanying uncertainty should be reported, how models should be evaluated, and what methodologies work well in which situations. Prediction efforts should start with a key public health objective. For dengue, a global public health problem that can affect millions of people per year, prediction of epidemics is a valuable objective because local incidence varies substantially from year to year. If dengue epidemics could be predicted, interventions such as vector control and preparing healthcare systems for a surge of patients, could reduce the impact. The accuracy and uncertainty of those predictions would have a direct effect on the cost-effectiveness of those measures.

Here we evaluated the forecasting capability of SARIMA models for predicting dengue incidence in Mexico and 17 Mexican states up to 6 months into the future. Importantly, this is a mature statistical framework used by many other dengue forecasters^11^,^14^,^15^,^18^,^19^,^24^,^25^,^27^,^35^,^37^,^40^,^43^,^45^,^46^,^47^. We compared 39 different models from the most simplistic, expecting future incidence to be the average of past incidence, to relatively complex seasonal autoregressive models incorporating weather covariates.

**Figure 6:**
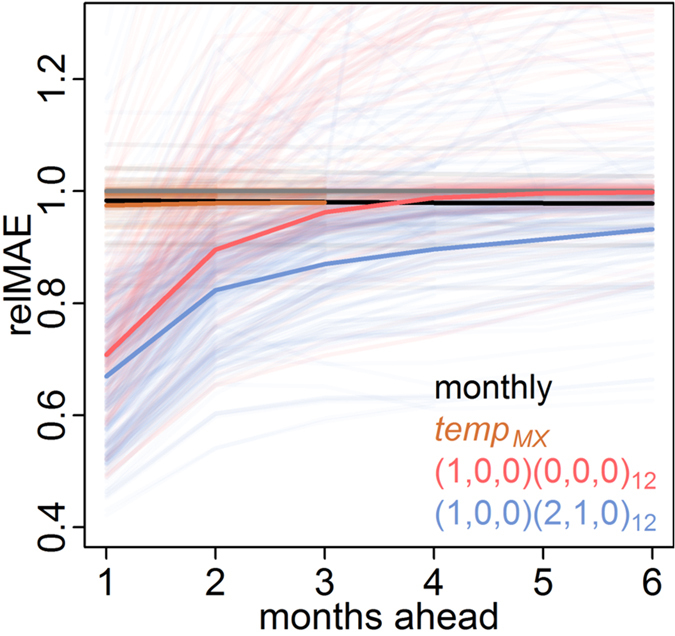
Prediction horizons. The relative MAEs compared to the constant model (grey line) are shown in transparent lines for all locations at all prediction horizons. Models including only weather covariates are shown in orange, models containing only short-term autocorrelation are shown in red, and models containing both short-term autocorrelation and seasonality (using weather variables or seasonal autocorrelation) are shown in blue. The average relative MAE across all locations is shown in solid lines for four key representative models.

For each state and all prediction horizons, models including seasonality via a naïve monthly term or weather variables were more accurate than the constant model (Fig. 6). For some states, weather data slightly improved prediction at short time scales compared to the naïve seasonal model. These differences may result from ecological heterogeneities that lead to different transmission dynamics in different places^54^. Alternatively, they may represent slightly better correlated covariates from a selection of highly correlated lagged variables that capture seasonality or confound seasonality with autocorrelation. This potentially leads to overfitting. In Michoacán for example, the local model including temperature performed poorly in the out-of-sample validation period. Regardless of their cause, the improvements resulting from inclusion of weather variables were generally very slight, and only Jalisco had a clear improvement in relative accuracy in both the evaluation and validation datasets.

Models including short-term autocorrelation meanwhile had improved accuracy for short-term prediction compared to seasonal models (Fig. 6). However, the strongest performance was attained by combining short-term autocorrelation and seasonality. The strong performance of the (1,0,0)(2,1,0)_12_ and (1,0,0)(3,1,0)_12_ models across states indicates that this type of model is a good reference model for dengue prediction. These models captured the short-term autocorrelation, but also a long-term negative autocorrelation of seasonal differences with coefficients of declining strength at increasing lags. In more general terms, this means that following a season with a substantial increase in cases, there will likely be fewer cases in subsequent seasons with that dampening decreasing over time. This makes sense for dengue as heterologous and homologous immunity can transiently limit transmissibility following large outbreaks^55^ and endemic dengue exhibits clear multiyear periodicity^56^.

While we have used a set of common climate variables in our analysis, considering additional climate covariates could provide additional information or benefits in predictive accuracy. Numerous studies have shown correlations between dengue cases and temperature, relative humidity, and precipitation as indicated in the introduction as well as absolute humidity, more recently^57^,^58^. Since temperature, relative humidity, and precipitation are closely related to absolute humidity, we do not expect that absolute humidity or other highly correlated climate variables will yield significant improvements to the model performance presented here. The framework nonetheless provides a straightforward and comprehensive procedure for assessing the predictive value of these variables or any others.

While this investigation revealed clear differences between models, it also revealed clear limitations to all the assessed models. At the 1 month horizon, R^2^ for the optimal models rarely exceeded 0.8 and accuracy rapidly declined for longer horizons. Furthermore, although R^2^ and mean absolute error can be used to assess and compare model fit, they are not sufficient for determining public health utility. As shown in Fig. 4A, in many cases both the local and common models failed to predict major epidemics at a 3-month horizon. The uncertainty in the predictions shown in Fig. 5 and Figure S2 reflects this and is large enough that there can be little expected public health utility at prediction horizons beyond a month with these models alone.

These findings capture broader challenges in the field of infectious disease forecasting. Published models vary in both approach and results. As shown here, numerous approaches can yield similar results, yet there is still variability in results. That variability may capture true heterogeneity or it may represent practically insignificant differences between essentially very similar models. For dengue model development, models incorporating both autocorrelation and seasonality, such as the (1,0,0)(2,1,0)_12_ model, should always be used as a baseline for comparison. Despite not including any data other than historical dengue data, this model captured a substantial amount of variation in dengue incidence. It therefore provides an easy benchmark for identifying substantive advances in dengue prediction.

We did not explore an exhaustively large space of possible prediction models, choosing instead to focus on the SARIMA framework. These models are widely used and accepted as robust tools for time-series prediction, but their limitations for dengue are clear. Other types of model frameworks and data could and should be explored for use instead of or in combination with these types of models. Other approaches used for forecasting dengue incidence include a variety of generalized linear models, machine learning techniques, and incorporation of novel data sources^12^,^13^,^16^,^19^,^20^,^21^,^22^,^23^,^26^,^27^,^28^,^31^,^33^,^34^,^36^,^37^,^38^,^39^,^41^,^43^,^44^,^47^. Mechanistic approaches may also be required specifically to capture changes in human immunity in response to past infection^5^,^6^,^55^. Other types of data such as vector surveillance data and DENV serotype- and genotype-specific data may also be critical. Furthermore, the inclusion of information from disparate (and complementary) disease-related data sources, such as cloud-based electronic health records, Twitter micro-blogs, and Google searches has been shown to improve short-term influenza forecasts substantially^59^.

With new approaches, robust benchmarking, testing, and validation will be critical to advancing the dengue forecasting. Here, we used 28 years of historical data to establish three datasets: training, evaluation, and validation. For every model considered, forecasts were made stepwise for every month of the evaluation period. Performance over this period was used to select two models, the common, baseline model and a local short-term model. The relative performance of these two models was then evaluated on the independent validation dataset. This framework follows the standard for evaluating prediction models as developed for machine learning^60^. Modeling efforts that leave out or combine the testing and validation components can lead to over-fitted models that perform poorly when used for prospective prediction, the ultimate goal. For example, if the original Google Flu Trends (GFT) work on influenza prediction^61^ used a similar framework, the benefits and limitations of the approach could have been better understood. Several years later, it became clear that at key times the GFT-based predictions were substantially off and that a simple autoregressive model had comparable performance^62^,^63^.

This study, like most, was restricted to archived historical data. While we took care to limit every forecast to data from before that time point and separate the evaluation and validation datasets, this is not the same as making truly prospective forecasts. Real-time forecasting is the objective and requires an extensive operational pipeline that can take raw real-time surveillance data (usually only partially observed) and produce accurate, calibrated forecasts with appropriately stated uncertainty. The work presented here does not meet these criteria. Rather, it helps to build the foundation for those systems, as a robust assessment of forecast performance on historical data is the best indicator of prospective performance.

We anticipate that the next decade will bear witness to a rapid maturation of techniques, methods, and frameworks that will increase our capacity to generate accurate, timely, and appropriately calibrated disease forecasts. With the growth of this field it is essential to establish robust methods for forecast development and evaluation. The first step in this process is to work with public health decision makers to identify appropriate targets and data. Next, as shown here, the data need to be clearly defined and separated into development and validation datasets comparing predictions to at least one baseline model that is not completely naïve. Model development coupled with systematic evaluation is critical for the future implementation of infectious disease forecasting to address real-time public health needs.

## Additional Information

**How to cite this article**: Johansson, M. A. *et al*. Evaluating the performance of infectious disease forecasts: A comparison of climate-driven and seasonal dengue forecasts for Mexico. *Sci. Rep.* **6**, 33707; doi: 10.1038/srep33707 (2016).

## Supplementary Material

Supplementary Information
