# Supplementary material for: Evaluating the performance of infectious disease forecasts: A comparison of climate-driven and seasonal dengue forecasts for Mexico
Source: Sci Rep. 2016 Sep 26;6:33707. doi: 10.1038/srep33707 (PMC5036038; doi:10.1038/srep33707)
Supplement: Supplementary Information [file srep33707-s1.pdf]

Evaluating the performance of infectious disease forecasts: A comparison of climate-driven and seasonal dengue forecasts for Mexico

Michael A. Johansson[1,2], Nicholas G. Reich[3], Aditi Hota[4], John S. Brownstein[4,5], Mauricio Santillana[4,5,6]

AUTHOR AFFILIATIONS

[1] Dengue Branch, Division of Vector-Borne Diseases, Centers for Disease Control and Prevention, San Juan, Puerto Rico

[2] Center for Communicable Disease Dynamics, Harvard T. H. Chan School of Public Health, Boston, Massachusetts

[3] Department of Biostatistics and Epidemiology, University of Massachusetts, Amherst, Massachusetts

[4] Computational Health Informatics Program, Boston Children's Hospital, Boston, Massachusetts

[5] Department of Pediatrics, Harvard Medical School, Boston, Massachusetts

[6] J.A. Paulson School of Engineering and Applied Sciences, Harvard University, Cambridge, MA

SUPPLEMENTARY FILES

Supplementary Figure 1. State-level forecast metrics. In each panel, state-level incidence is shown during the training period (1985–1989) and evaluation period (1990–2007) (A). For each of 39 models considered, the MAE (B) and R^2 (C) values for prospective forecasts over the entire evaluation period are shown for each prediction horizon (dark red to yellow, corresponds to 1 to 6 months). For models including lagged weather covariates, forecasts were not possible at prediction horizons beyond the lag and are not shown.

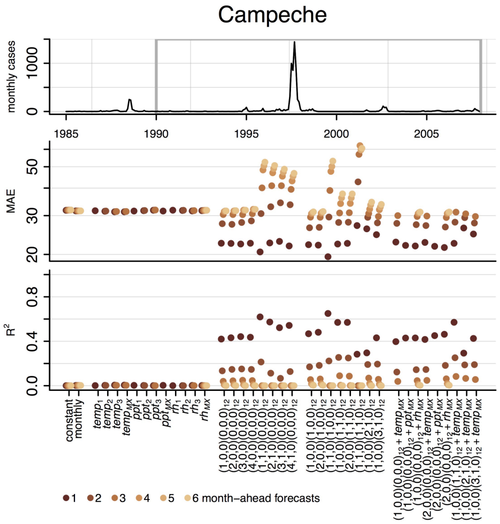
Campeche
monthly cases
MAE
R^2
1 2 3 4 5 6 month–ahead forecasts

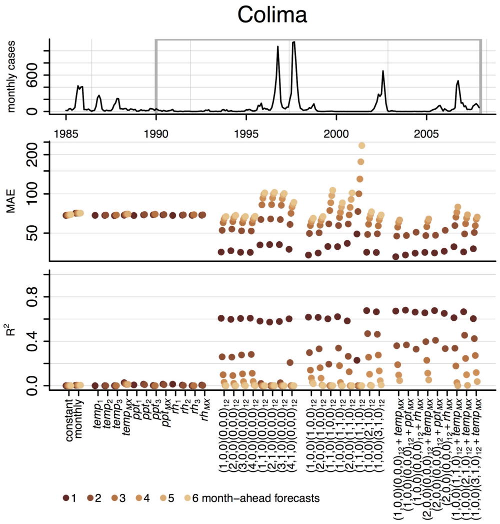
Colima
monthly cases
MAE
R^2
1 2 3 4 5 6 month–ahead forecasts

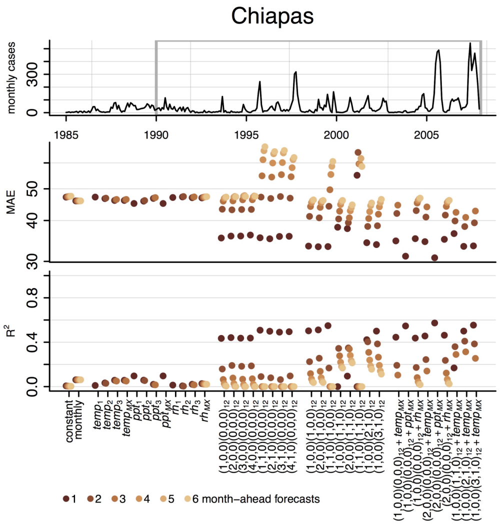
Chiapas
monthly cases
MAE
R^2
1 2 3 4 5 6 month–ahead forecasts

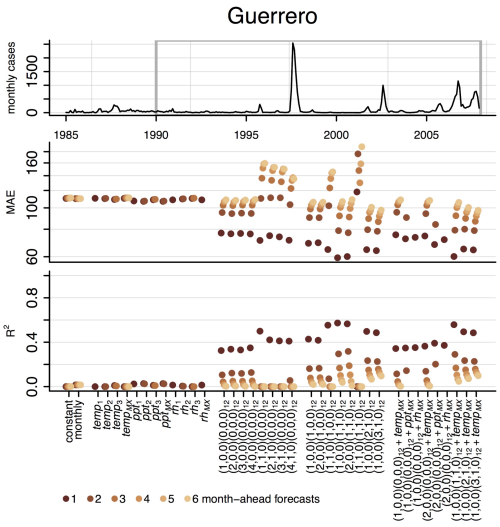
Guerrero
monthly cases
MAE
R^2
1 2 3 4 5 6 month–ahead forecasts

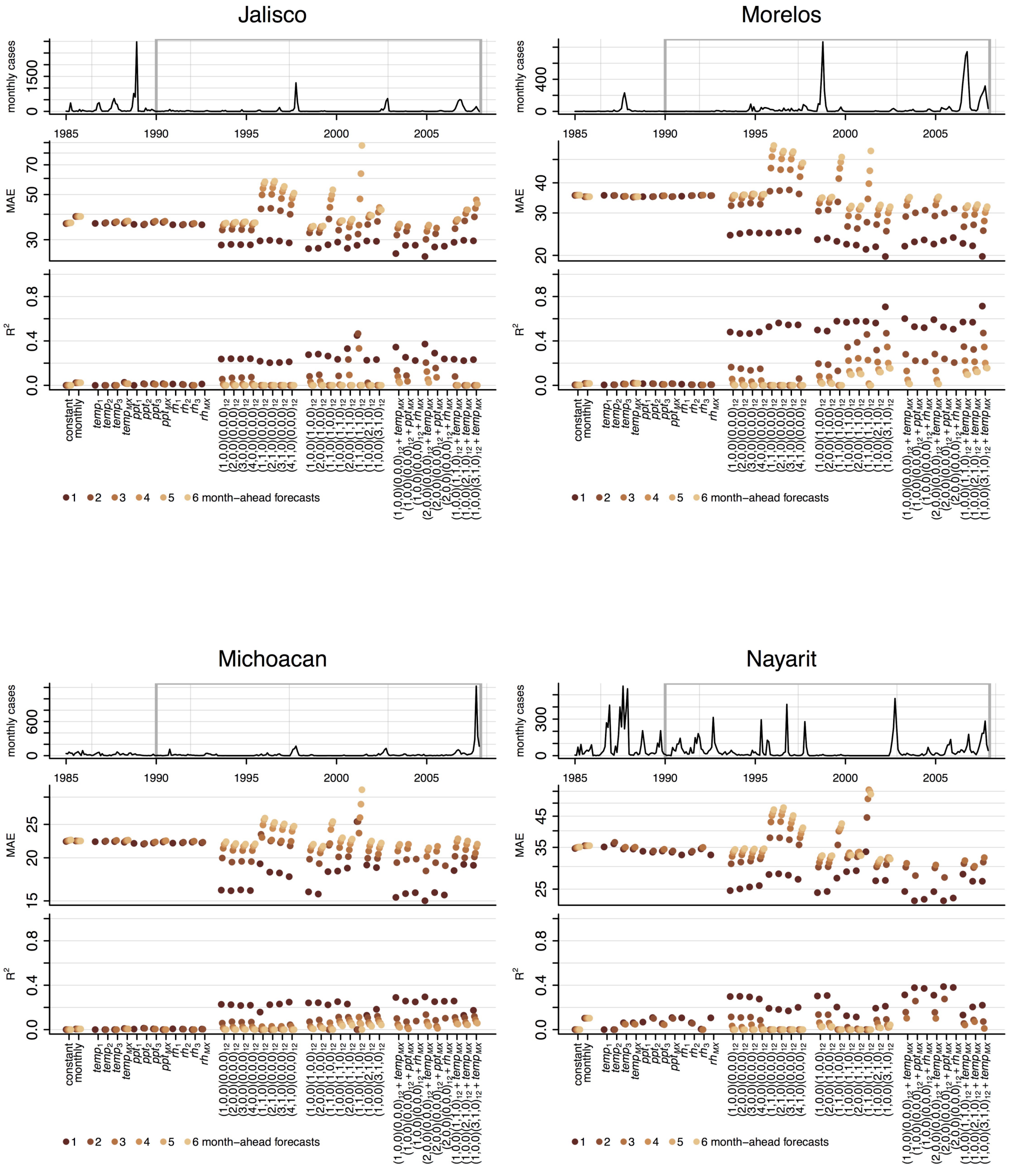
Jalisco
Morelos
Michoacan
Nayarit
monthly cases
MAE
R²
1985
1990
1995
2000
2005
1 2 3 4 5 6 month–ahead forecasts

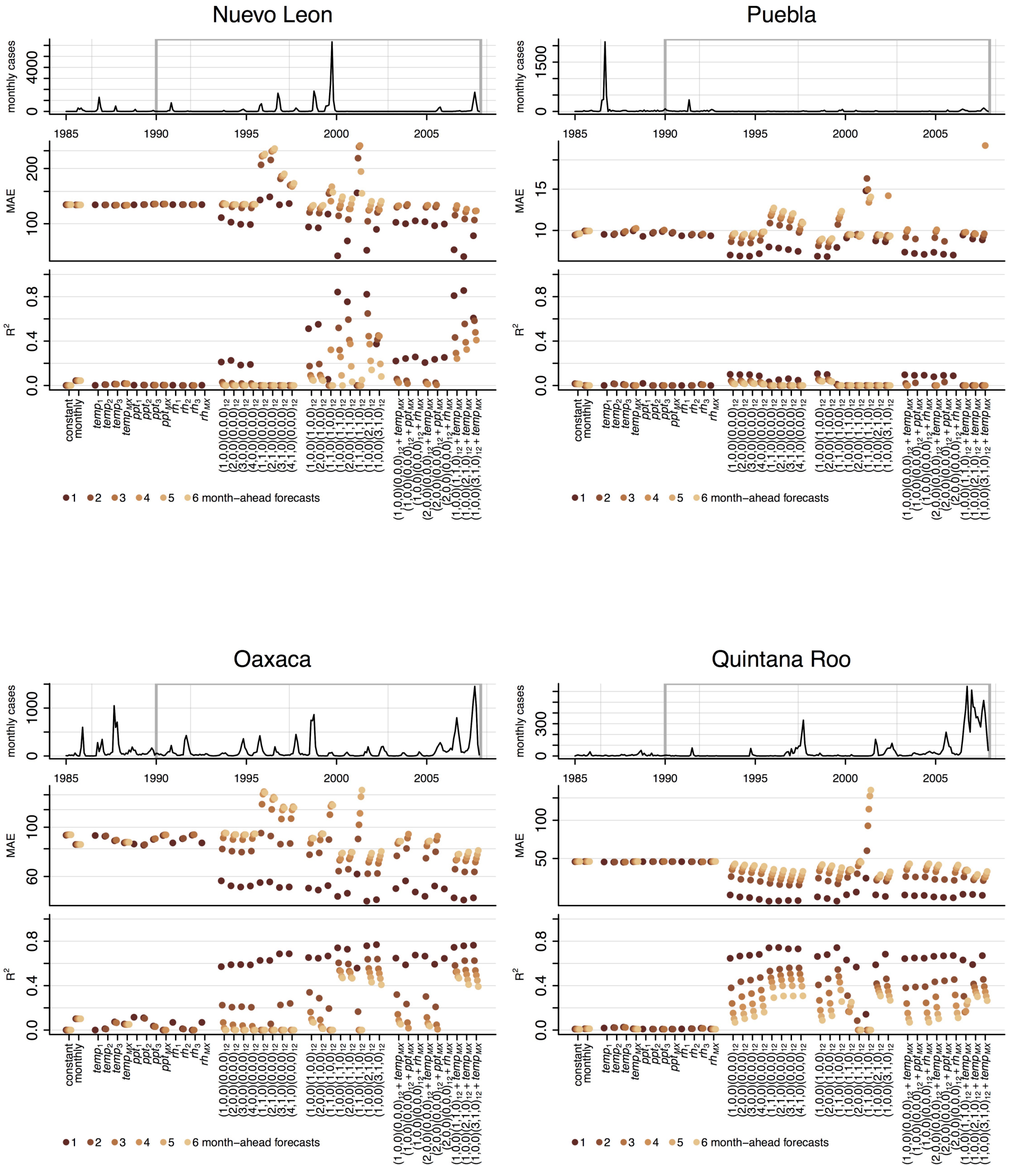

Nuevo Leon
Puebla
Oaxaca
Quintana Roo
monthly cases
MAE
R2
1985
1990
1995
2000
2005
constant
monthly
1 2 3 4 5 6 month–ahead forecasts

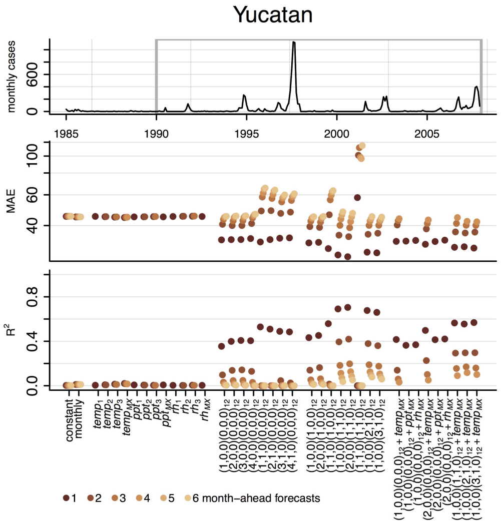

Yucatan
monthly cases
0
600
1985
1990
1995
2000
2005
MAE
40
60
100
R²
0.0
0.4
0.8
constant
monthly
1 2 3 4 5 6 month–ahead forecasts

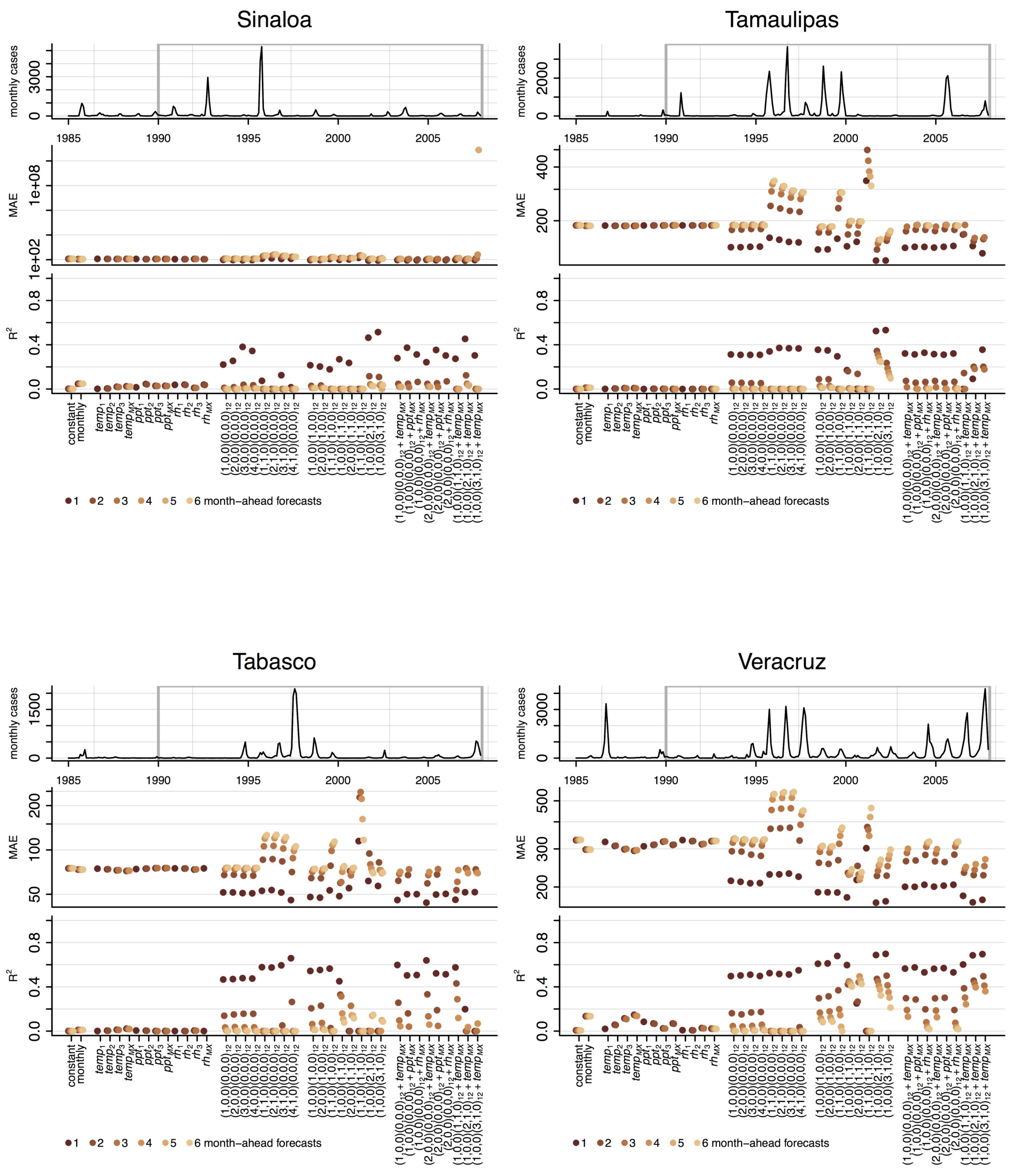

Supplementary Figure 2. Prospective predictions with uncertainty by locations. Forecasts from common and local models for each location are shown with 95% Confidence Intervals for each prediction horizon (1-6 months). Reported dengue incidence is shown as a black, dashed line.

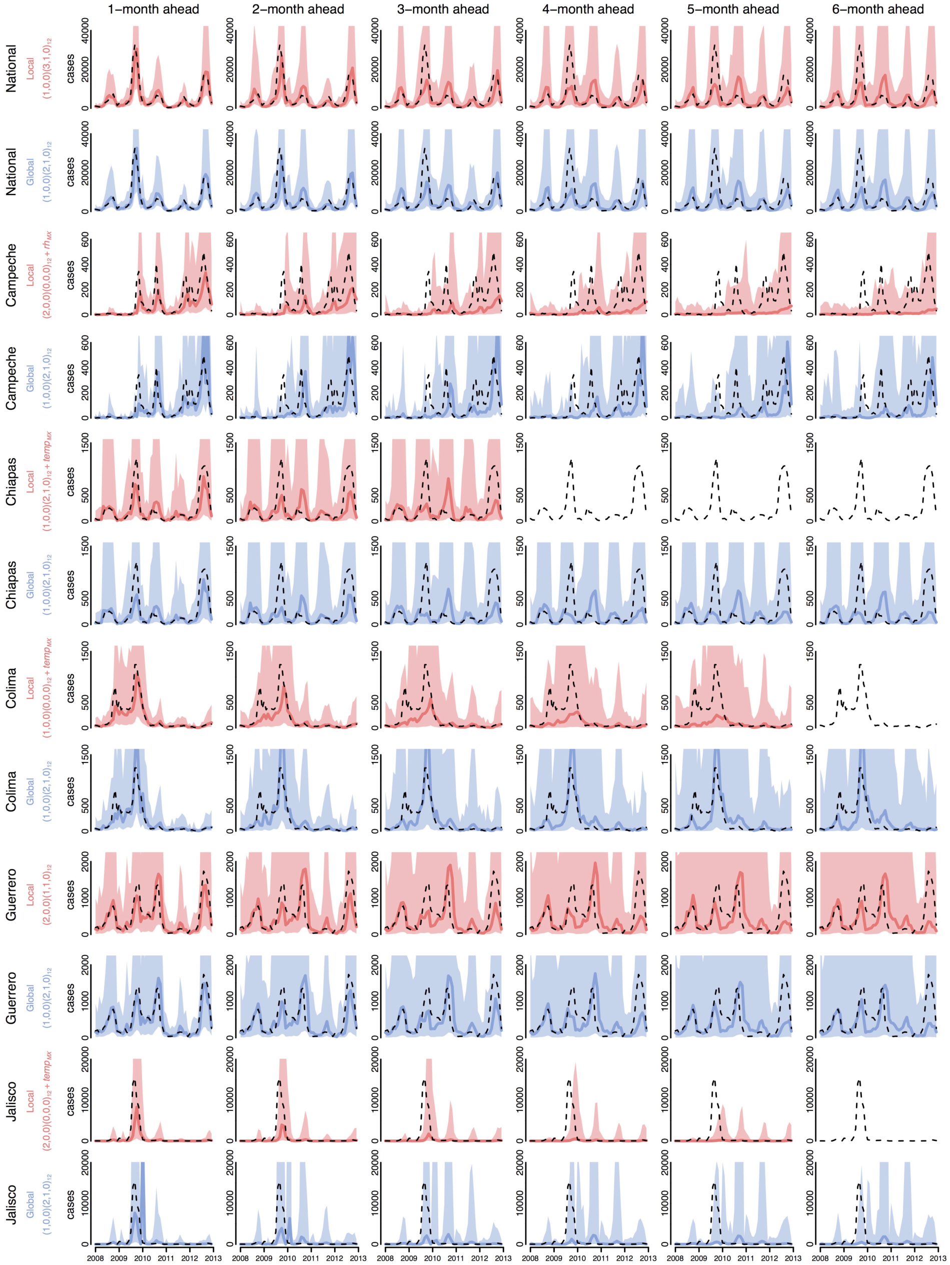
1−month ahead
2−month ahead
3−month ahead
4−month ahead
5−month ahead
6−month ahead
National
Local
(1,0,0)(3,1,0)12
National
Global
(1,0,0)(2,1,0)12
Campeche
Local
(2,0,0)(0,0,0)12 + rhMX
Campeche
Global
(1,0,0)(2,1,0)12
Chiapas
Local
(1,0,0)(2,1,0)12 + tempMX
Chiapas
Global
(1,0,0)(2,1,0)12
Colima
Local
(1,0,0)(0,0,0)12 + tempMX
Colima
Global
(1,0,0)(2,1,0)12
Guerrero
Local
(2,0,0)(1,1,0)12
Guerrero
Global
(1,0,0)(2,1,0)12
Jalisco
Local
(2,0,0)(0,0,0)12 + tempMX
Jalisco
Global
(1,0,0)(2,1,0)12
cases
2008
2009
2010
2011
2012
2013

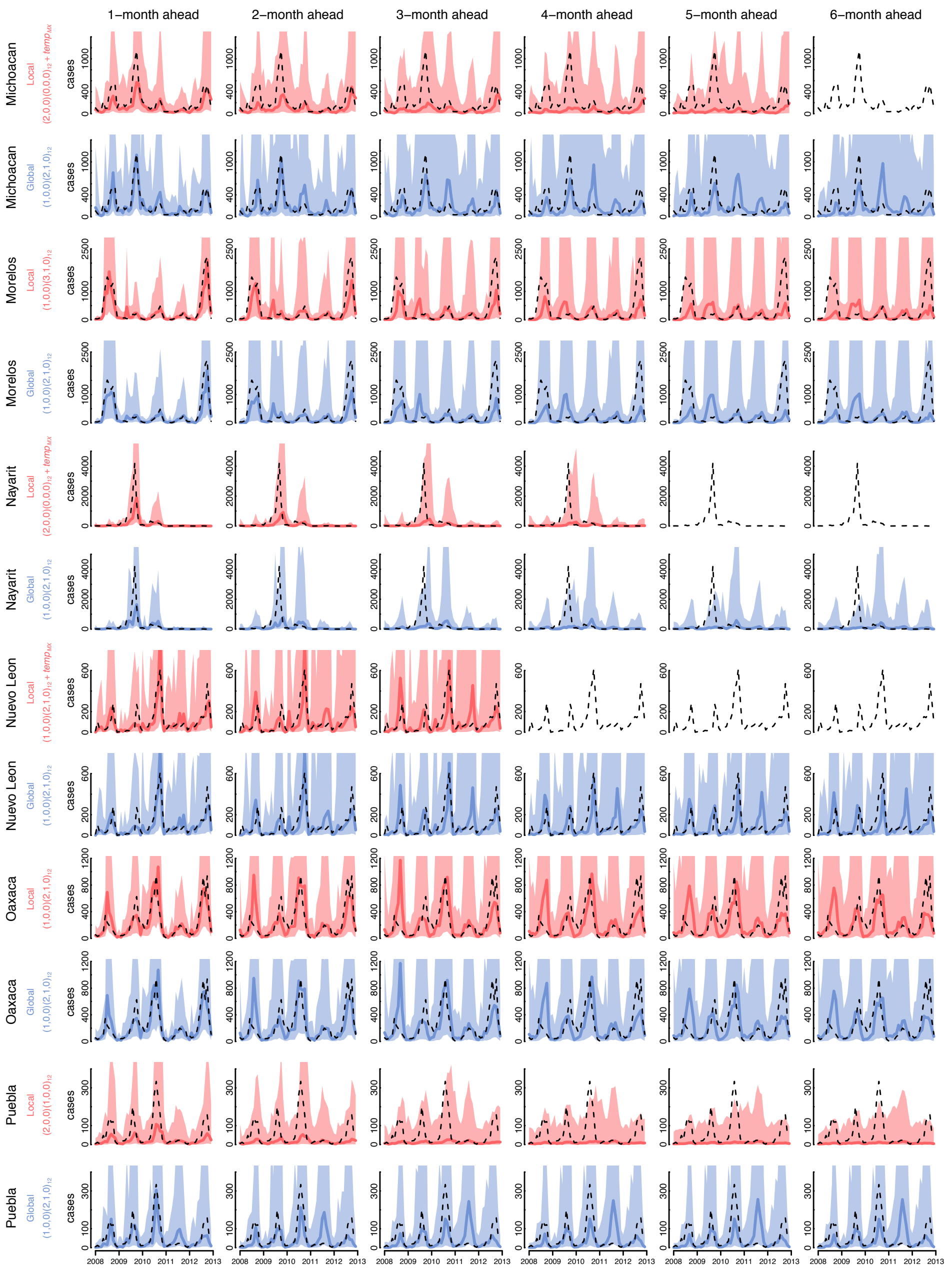
1−month ahead
2−month ahead
3−month ahead
4−month ahead
5−month ahead
6−month ahead
Michoacan
Local
Michoacan
Global
Morelos
Local
Morelos
Global
Nayarit
Local
Nayarit
Global
Nuevo Leon
Local
Nuevo Leon
Global
Oaxaca
Local
Oaxaca
Global
Puebla
Local
Puebla
Global
cases
2008
2009
2010
2011
2012
2013

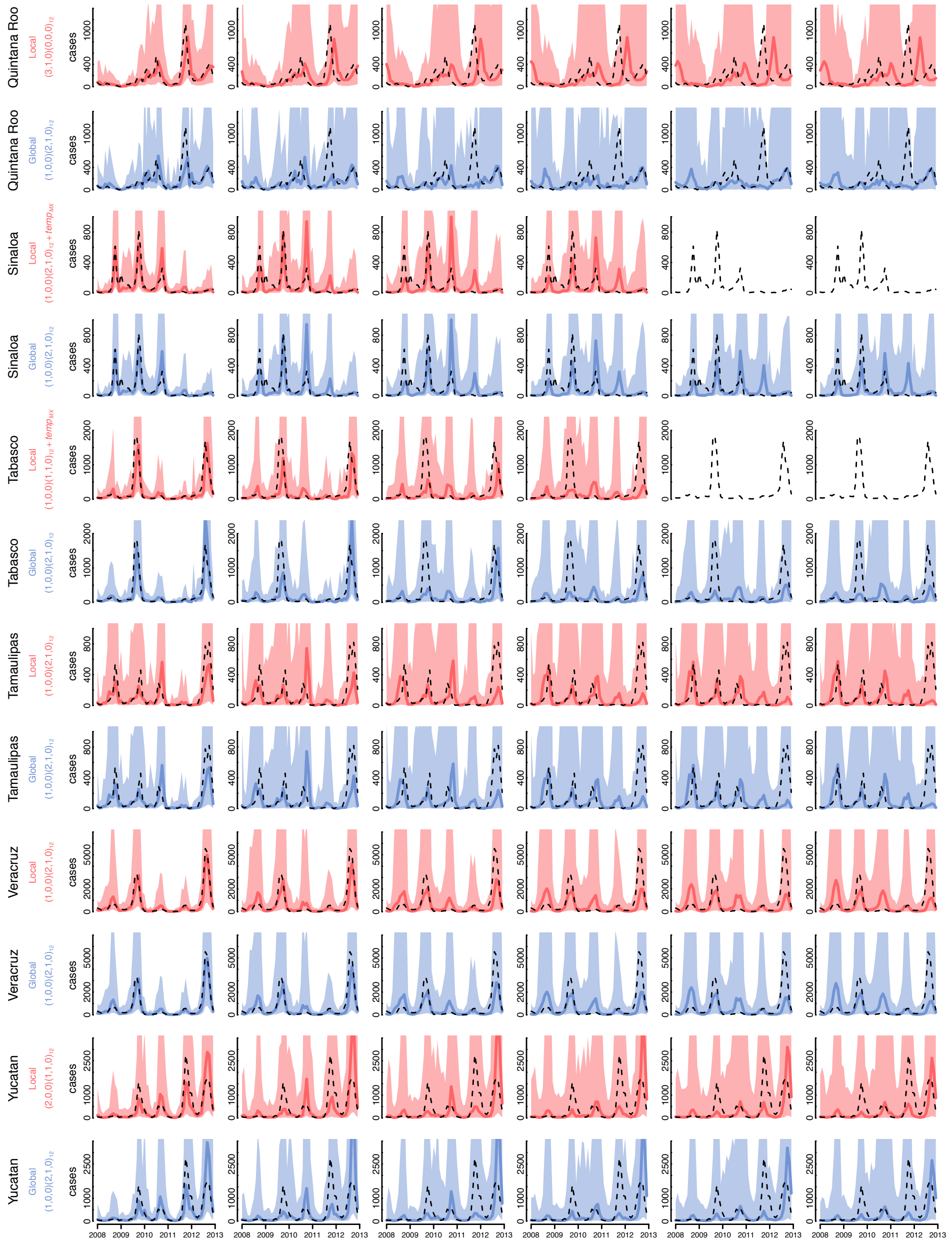

Supplementary Table. Model coefficients. For each location, this table reports the coefficients, corresponding confidence intervals, partial log likelihood (pll), and number of estimated parameters (k) for the common model (optimal model for across all locations and horizons) and location-specific local models (optimal model for local 1-3 month ahead forecasts). The **α** and **φ** coefficients correspond to the short-term and seasonal

autoregressive coefficients in equation 1. $\mathbf{B_0}$ is the intercept for non-differenced local models including covariates and $\boldsymbol{\beta_1}$ is the coefficient for the covariate, when present. Lagged covariates included relative humidity for Campeche and average temperature for Chiapas, Colima, Jalisco, Michoacán, Nayarit, Nuevo Leon, Sinaloa, and Tabasco.

		α_1		α_2		α_3		ϕ_1		ϕ_2		ϕ_3		β_0		β_1			
		est.	95% CI	est.	95% CI	est.	95% CI	est.	95% CI	est.	95% CI	est.	95% CI	est.	95% CI	est.	95% CI	pll	k
National	common	0.850	(0.791, 0.908)	-	-	-	-	-0.583	(-0.688, -0.477)	-0.225	(-0.329, -0.121)	-	-	-	-	-	-	-273.5	3
	local	0.876	(0.823, 0.929)	-	-	-	-	-0.666	(-0.770, -0.562)	-0.387	(-0.502, -0.271)	-0.251	(-0.351, -0.151)	-	-	-	-	-257.9	4
Campeche	common	0.852	(0.795, 0.909)	-	-	-	-	-0.637	(-0.743, -0.531)	-0.228	(-0.330, -0.126)	-	-	-	-	-	-	-440.9	3
	local	0.793	(0.686, 0.901)	0.074	(-0.034, 0.182)	-	-	-	-	-	-	-	-	-0.252	(-1.712, 1.208)	0.029	(0.011, 0.047)	-429.9	4
Chiapas	common	0.704	(0.626, 0.783)	-	-	-	-	-0.475	(-0.577, -0.373)	-0.286	(-0.384, -0.188)	-	-	-	-	-	-	-442.9	3
	local	0.702	(0.624, 0.781)	-	-	-	-	-0.473	(-0.577, -0.370)	-0.283	(-0.383, -0.183)	-	-	-	-	0.037	(-0.045, 0.119)	-426.1	4
Colima	common	0.909	(0.864, 0.955)	-	-	-	-	-0.565	(-0.667, -0.463)	-0.390	(-0.490, -0.290)	-	-	-	-	-	-	-404.5	3
	local	0.913	(0.870, 0.956)	-	-	-	-	-	-	-	-	-	-	0.079	(-1.43, 1.588)	0.116	(0.068, 0.163)	-381.3	3
Guerrero	common	0.843	(0.784, 0.902)	-	-	-	-	-0.495	(-0.595, -0.395)	-0.221	(-0.311, -0.131)	-	-	-	-	-	-	-400.0	3
	local	0.706	(0.598, 0.814)	0.143	(0.036, 0.251)	-	-	-0.411	(-0.501, -0.321)	-	-	-	-	-	-	-	-	-422.8	3
Jalisco	common	0.725	(0.650, 0.799)	-	-	-	-	-0.511	(-0.613, -0.409)	-0.233	(-0.329, -0.137)	-	-	-	-	-	-	-557.5	3
	local	0.621	(0.517, 0.725)	0.206	(0.103, 0.310)	-	-	-	-	-	-	-	-	-1.821	(-3.181, -0.460)	0.196	(0.145, 0.247)	-556.7	4
Michoacan	common	0.782	(0.714, 0.851)	-	-	-	-	-0.516	(-0.618, -0.414)	-0.212	(-0.306, -0.118)	-	-	-	-	-	-	-447.2	3
	local	0.634	(0.530, 0.737)	0.250	(0.146, 0.354)	-	-	-	-	-	-	-	-	-0.535	(-1.783, 0.714)	0.132	(0.093, 0.171)	-452.1	4
Morelos	common	0.783	(0.715, 0.852)	-	-	-	-	-0.539	(-0.641, -0.437)	-0.283	(-0.385, -0.181)	-	-	-	-	-	-	-433.9	3
	local	0.835	(0.772, 0.898)	-	-	-	-	-0.595	(-0.697, -0.493)	-0.475	(-0.585, -0.365)	-0.323	(-0.419, -0.227)	-	-	-	-	-405.6	4
Nayarit	common	0.792	(0.725, 0.858)	-	-	-	-	-0.567	(-0.671, -0.463)	-0.307	(-0.405, -0.209)	-	-	-	-	-	-	-468.7	3
	local	0.772	(0.664, 0.880)	0.064	(-0.044, 0.171)	-	-	-	-	-	-	-	-	0.404	(-0.874, 1.682)	0.101	(0.052, 0.150)	-478.9	4
Nuevo Leon	common	0.743	(0.668, 0.817)	-	-	-	-	-0.524	(-0.626, -0.422)	-0.187	(-0.285, -0.089)	-	-	-	-	-	-	-488.2	3
	local	0.747	(0.672, 0.821)	-	-	-	-	-0.512	(-0.617, -0.406)	-0.178	(-0.28, -0.076)	-	-	-	-	-0.069	(-0.146, 0.007)	-468.6	4
Oaxaca	common	0.834	(0.773, 0.895)	-	-	-	-	-0.649	(-0.747, -0.551)	-0.286	(-0.372, -0.200)	-	-	-	-	-	-	-358.8	3
	local	0.834	(0.773, 0.895)	-	-	-	-	-0.649	(-0.747, -0.551)	-0.286	(-0.372, -0.200)	-	-	-	-	-	-	-358.8	3
Puebla	common	0.502	(0.406, 0.598)	-	-	-	-	-0.418	(-0.520, -0.316)	-0.207	(-0.305, -0.109)	-	-	-	-	-	-	-495.6	3
	local	0.554	(0.445, 0.664)	0.144	(0.038, 0.250)	-	-	0.280	(0.171, 0.390)	-	-	-	-	1.756	(1.215, 2.297)	-	-	-507.9	4
Quintana Roo	common	0.823	(0.760, 0.885)	-	-	-	-	-0.619	(-0.719, -0.519)	-0.340	(-0.438, -0.242)	-	-	-	-	-	-	-434.8	3
	local	-0.198	(-0.304, -0.092)	-0.097	(-0.205, 0.011)	-0.128	(-0.234, -0.022)	-	-	-	-	-	-	-	-	-	-	-421.4	3
Sinaloa	common	0.749	(0.674, 0.823)	-	-	-	-	-0.584	(-0.686, -0.482)	-0.298	(-0.396, -0.200)	-	-	-	-	-	-	-460.8	3
	local	0.748	(0.672, 0.824)	-	-	-	-	-0.584	(-0.688, -0.480)	-0.299	(-0.399, -0.199)	-	-	-	-	-0.007	(-0.101, 0.088)	-443.7	4
Tabasco	common	0.857	(0.800, 0.914)	-	-	-	-	-0.597	(-0.699, -0.495)	-0.318	(-0.414, -0.222)	-	-	-	-	-	-	-424.8	3
	local	0.810	(0.743, 0.877)	-	-	-	-	-0.398	(-0.496, -0.300)	-	-	-	-	-	-	0.120	(0.042, 0.198)	-428.0	3
Tamaulipas	common	0.802	(0.735, 0.869)	-	-	-	-	-0.511	(-0.612, -0.409)	-0.308	(-0.406, -0.210)	-	-	-	-	-	-	-511.9	3
	local	0.802	(0.735, 0.869)	-	-	-	-	-0.511	(-0.612, -0.409)	-0.308	(-0.406, -0.210)	-	-	-	-	-	-	-511.9	3
Veracruz	common	0.777	(0.708, 0.845)	-	-	-	-	-0.603	(-0.701, -0.505)	-0.398	(-0.496, -0.300)	-	-	-	-	-	-	-434.9	3
	local	0.777	(0.708, 0.845)	-	-	-	-	-0.603	(-0.701, -0.505)	-0.398	(-0.496, -0.300)	-	-	-	-	-	-	-434.9	3
Yucatan	common	0.801	(0.736, 0.865)	-	-	-	-	-0.541	(-0.643, -0.439)	-0.311	(-0.409, -0.213)	-	-	-	-	-	-	-478.2	3
	local	0.736	(0.627, 0.846)	0.059	(-0.051, 0.169)	-	-	-0.404	(-0.500, -0.308)	-	-	-	-	-	-	-	-	-496.9	3
